# Dose prescription with spatial uncertainty for peripheral lung SBRT

**DOI:** 10.1002/acm2.12504

**Published:** 2018-12-14

**Authors:** James L. Bedford, Irena Blasiak‐Wal, Vibeke N. Hansen

**Affiliations:** ^1^ Joint Department of Physics The Institute of Cancer Research The Royal Marsden NHS Foundation Trust London UK; ^2^ Present address: Odense University Hospital Denmark

**Keywords:** dose prescription, probabilistic treatment planning, SBRT

## Abstract

Current clinical practice is to prescribe to 95% of the planning target volume (PTV) in 4D stereotactic body radiotherapy (SBRT) for lung. Frequently the PTV margin has a very low physical density so that the internal target volume (ITV) receives an unnecessarily high dose. This study investigates the alternative of prescribing to the ITV while including the effects of positional uncertainties. Five patients were retrospectively studied with volumetric modulated arc therapy treatment plans. Five plans were produced for each patient: a static plan prescribed to PTV *D*_95%_, three probabilistic plans prescribed to ITV *D*_95%_ and a static plan re‐prescribed to ITV *D*_95%_ after inverse planning. For the three probabilistic plans, the scatter kernel in the dose calculation was convolved with a spatial uncertainty distribution consisting of either a uniform distribution extending ±5 mm in the three orthogonal directions, a distribution consisting of delta functions at ±5 mm, or a Gaussian distribution with standard deviation 5 mm. Median ITV *D*_50%_ is 23% higher than the prescribed dose for static planning and only 10% higher than the prescribed dose for prescription to the ITV. The choice of uncertainty distribution has less than 2% effect on the median ITV dose. Re‐prescribing a static plan and evaluating with a probabilistic dose calculation results in a median ITV *D*_95%_ which is 1.5% higher than when planning probabilistically. This study shows that a robust probabilistic approach to planning SBRT lung treatments results in the ITV receiving a dose closer to the intended prescription. The exact form of the uncertainty distribution is not found to be critical.

## 1 INTRODUCTION

Stereotactic body radiotherapy (SBRT) has been shown to be an effective treatment for small lung lesions, such as oligometastases.^1^, ^2^ Typically, the gross tumor volume (GTV) is contoured on several phases of a 4DCT scan, to yield an internal target volume (ITV) encompassing the tumor throughout all breathing phases. A planning target volume (PTV) consisting of the ITV plus 5 mm is then used for treatment planning. Patients treated with SBRT on linear accelerators are always imaged with cone‐beam CT (CBCT) immediately before each treatment fraction, so that the spatial uncertainty of the ITV is low. The PTV margin accounts for any residual positional uncertainty after registration of the CBCT to the planning CT, together with any tumor delineation uncertainty.^3^

Treatment plans for this technique are invariably prescribed such that 95% of the PTV receives the prescribed dose. The planning target margin often lies in low‐density lung material, so that the ITV itself receives a much higher dose than the edge of the PTV.^4^, ^5^ In an ablative context, this is not in itself problematic, but clinical protocols normally specify a maximum dose of around 140% of the prescribed dose, which can then be difficult to achieve. Furthermore, normal tissue tolerances can be exceeded due to the excessively high dose, creating difficulties in treatment planning and the risk of normal tissue toxicity.

A more satisfactory approach is to prescribe directly to the ITV. This volume is where the lesion itself is located throughout the breathing cycle, and it has a density much closer to that of water than the surrounding lung tissue. Prescribing to this volume therefore ensures that the lesion itself receives the correct dose, without over‐prescription due to the influence of low‐density areas surrounding it. However, simply prescribing to a static ITV located centrally within the PTV does not account for the spatial uncertainty in the ITV. Instead, the expected dose to the ITV in the presence of uncertainty should be used. This is carried out by convolving the delivered dose with the probability distribution of ITV position.

Some understanding of this approach has already been acquired in the field of probabilistic planning.^6^, ^7^ Furthermore, experience has also been obtained in the context of breathing motion compensation, as discussed by Lujan et al.^8^ These authors also investigate the outcome in the event that the actual probability density function (PDF) of position at the time of treatment does not match that at the time of planning.^9^ Other authors have also made similar investigations.^10^, ^11^ These studies have convolved the static dose distribution with the PDF of motion to obtain the expected dose distribution under respiration (dose convolution). This is appropriate because the motion or uncertainty distribution relates to a specific local region of the patient, while the overall patient position reflects no overall shift from planning. The primary dose calculation occurs once for the fixed geometry. An alternative is to convolve the incident fluence profile of each beam with the appropriate components of the PDF (fluence convolution).^12^, ^13^ This is computationally faster because the convolution is two‐dimensional rather than three‐dimensional. Zhang et al.^14^ and Trofimov et al.^15^ have extended this method to include nonrigid organ motion by evaluating the deformation during motion from multiple CT scans and incorporating this information into the planning process. For handling systematic setup errors, where the entire patient position varies in relation to the treatment beam, the primary fluence should be calculated to reflect this overall shift. In this case, fluence convolution is more appropriate, since the fluence is modified before ray‐tracing through the patient, and this modified fluence is then used in the calculation of the primary dose distribution (i.e., total energy released per unit mass, TERMA). This study assumes that the uncertainty distribution is localized to the ITV, so dose convolution is used. In practice, this is accomplished by convolving the scatter kernel with the uncertainty kernel, and then using this combined kernel for the dose calculation.

To a greater or lesser extent, it is possible to use the inverse planning process to compensate for the effects of spatial uncertainty.^8^, ^16^ This is the general concept of robust and probabilistic treatment planning,^17^, ^18^, ^19^, ^20^, ^21^, ^22^, ^23^ but this is not the primary goal of this study. Instead, the aim is to determine the target dose when prescribing directly to the ITV in the presence of spatial uncertainty. Accordingly, convolved dose distributions are used throughout inverse planning to avoid a sudden change in dose calculation at the end of inverse planning, while predominantly conformal arc beams are used to avoid compensation for the uncertainty.

This study aims to evaluate the dosimetric impact of prescribing directly to the ITV in lung SBRT treatment plans. Several possible distributions of ITV uncertainty are evaluated. Furthermore, to evaluate the situation if a clinic simply prescribes to the ITV without taking into account spatial uncertainty, inverse planning is carried out without the inclusion of spatial uncertainty, and then a final dose calculation is carried out with spatial uncertainty. It is expected that the study will lead to improved consistency of dose to the ITV, fewer compromises in the production of treatment plans, and avoidance of unnecessary dose to critical structures.

## 2 MATERIALS AND METHODS

Five lung SBRT patients were retrospectively studied. 4DCT scans were acquired using a Brilliance Big Bore CT scanner (Philips, Cleveland, OH, USA). The GTV was outlined on the 0% and 50% phases of the breathing cycle using Pinnacle^3^ (Philips, Madison, WI, USA), and combined to form an ITV. The ITV was normally just the envelope of the GTV volume on the 0% and 50% phases, but was checked visually using a movie presentation of the breathing motion to ensure that it encompassed the GTV at all times, and was edited if necessary. The uncertainty in the ITV contour, estimated as ±2 mm, was included in the PTV margin in the case of static planning, and by probability distributions in the case of probabilistic planning based on the ITV. The uncertainty was principally composed of (a) difficulty in assessing the extent of the gross tumor on the CT scan, (b) assessment of the motion of the lesion at the various phases of the breathing cycle, and (c) differences in breathing pattern between planning and delivery. Due to the difficulty in assessing the distribution of the uncertainty, its extent was taken to be of equal magnitude in each of the cardinal directions. Contouring was carried out in accord with UK stereotactic ablative radiotherapy Guidelines.^24^ Median ITV volume was 4.3 cm^3^ (range 1.6–14.9 cm^3^).

A series of volumetric modulated arc therapy treatment plans was constructed retrospectively for each patient using the AutoBeam (v5.8) in‐house treatment planning system. The isocenter was positioned at the center of the ITV in all cases, and the treatment beam consisted of a single gantry rotation from 178° to 182° with 4° control point spacing. The treatment plan was based on the 6 MV flattening filter‐free beam of a Versa HD accelerator (Elekta AB, Stockholm, Sweden).^25^ The control points were set into groups of 20° of gantry angle and the apertures in two out of every three groups were constrained to conform to the PTV, with no penumbra margin except 3 mm superiorly and inferiorly. In one out of every three control point groups, the apertures were modulated, with a minimum segment width of 15 mm. This hybrid approach was intended to give a robust treatment plan with mostly conformal segments, but with the scope to include some modulation to improve the dose distribution. The collimator angle was 2° in all cases.

**Figure 1:**
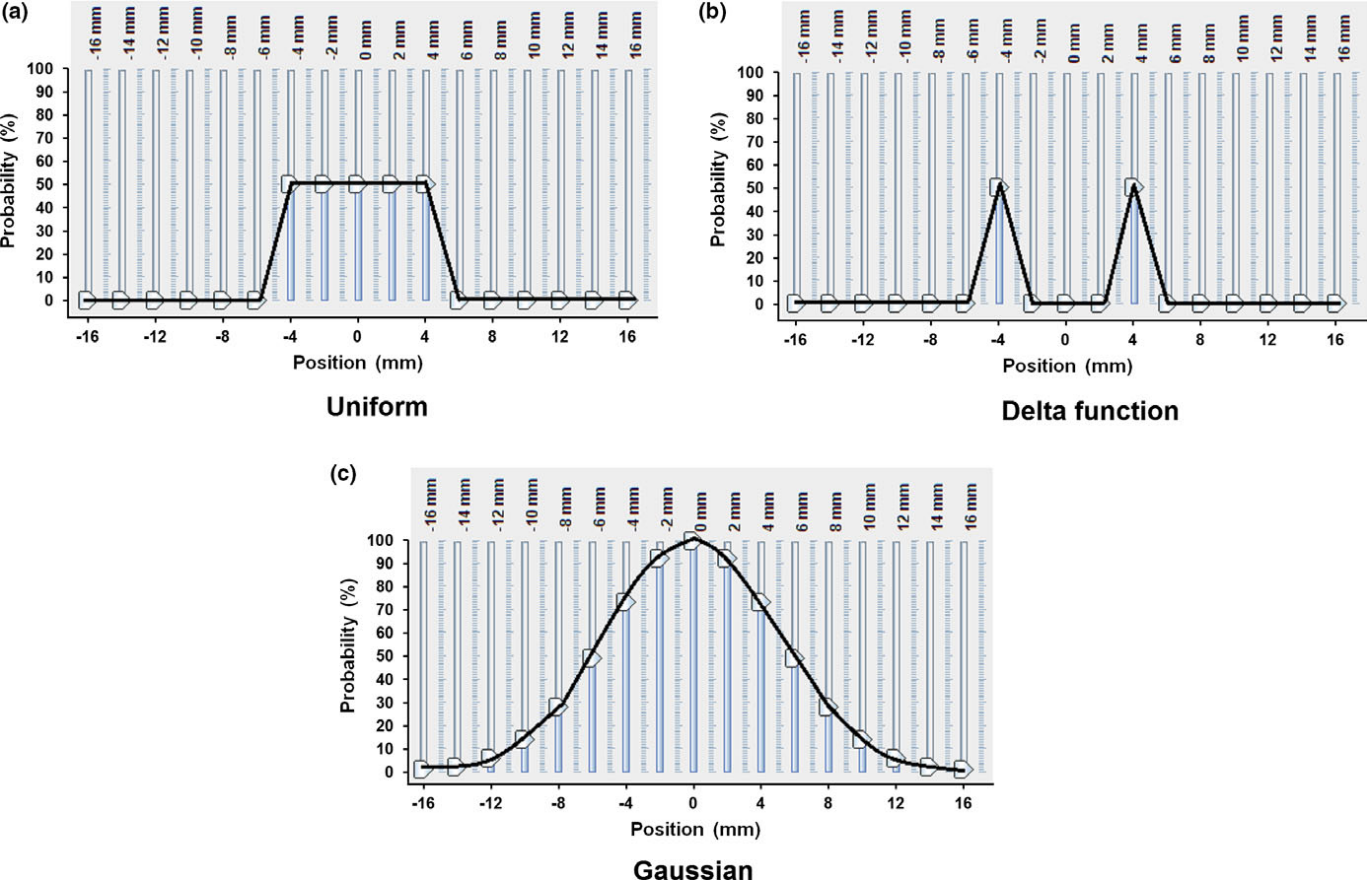
The three distributions used to describe spatial uncertainty. (a) Uniform distribution, (b) delta function distribution and (c) Gaussian distribution.

The prescription used in this study was 54 Gy in 3 fractions, but another fractionation scheme could equally have been used, with similar results. For each patient, a static plan was produced, with no spatial uncertainty distribution explicitly applied, and prescribed to the PTV as normal, such that PTV *D*_95%_ was 54 Gy in 3 fractions. In addition, three further plans were produced, each with a distribution of spatial uncertainty assigned to the ITV. The distributions were (a) a uniform distribution of position from −5 mm to +5 mm in each of the anterior‐posterior, left‐right and superior‐inferior directions, (b) a distribution consisting of delta functions at ±5 mm in each of the anterior‐posterior, left‐right and superior‐inferior directions, that is, assuming the tumor spent all of its time at the extremities of position, and (c) a Gaussian distribution with standard deviation 5 mm in each of the cardinal directions. Note that this latter distribution was somewhat wider than the other two due to its total width of around two standard deviations (Fig. 1). Each of the plans with spatial uncertainty was planned and prescribed to the ITV *D*_95%_. A final treatment plan for each patient investigated the effect of simply re‐prescribing to the ITV *D*_95%_. This plan was constructed and calculated as a static plan, prescribed to the PTV. After inverse planning, it was re‐prescribed to the ITV *D*_95%_ and, without changing the monitor units, recalculated using the Gaussian position distribution described above. The purpose of this plan was to determine the dose distribution in the presence of spatial uncertainty that resulted in the event that the plan was created normally and then simply re‐prescribed to the ITV.

Inverse planning was then accomplished using fluence optimization for the modulated control point groups, sequencing, and then aperture optimization. Both the fluence optimization and aperture optimization stages used iterative least squares.^26^ For fluence optimization, the iterative least squares method adjusted the fluence values, while for aperture optimization, it adjusted multileaf collimator positions. Dose was calculated using a fast convolution dose calculation^27^ on a grid of 2 mm × 2 mm × 2 mm in resolution.

Convolution was carried out in patient space. In principle, for a given point of interest, ***r***, the total dose, *D*, was given by^28^:

(1) $$D\left(r\right)=\int_{V^{\prime }}\phi \left(r^{\prime }\right)s\left(r-r^{\prime }\right)dV^{\prime }$$

where $\phi \left(r^{\prime }\right)$ was the primary fluence at point $r^{\prime }$, and $s\left(r-r^{\prime }\right)$ was a spatially invariant, polyenergetic scatter kernel. In this work, the total dose, *D*_*i*_(***r***) at a given point of interest, ***r***, was calculated by a discrete approximation:

(2) $$D_{i}\left(r\right)\approx \sum_{j=1}^{J}\phi \left(r_{i}+s_{j}\right)\sigma_{j},i=1\ldots I.$$

where ***s***_*j*_ was the position of the *j*th scatter point relative to the point of interest, and *σ*_*j*_ was the corresponding scatter contribution. For the discrete scatter kernel used in this work, *J* had a value of 16.

The effect of spatial uncertainty was included in the dose calculation by convolving the scatter kernel with a spatial uncertainty kernel, and then convolving the result with the primary dose distribution. The PDF of spatial uncertainty was specified as relative probabilities, at intervals of 2 mm, in the anterior‐posterior, left‐right and superior‐inferior directions, up to ±16 mm, giving 17 bins in each direction. Each combination of position in the three directions constituted a single point, ***u***_*k*_, in the PDF kernel, and the product of the individual histogram values for these positions yielded the corresponding weight, *ψ*_*k*_, of the kernel at that point. The motion kernel therefore consisted of *K *=* *17 × 17 × 17 = 4913 points and corresponding weights.

The uncertainty kernel was then convolved with the scatter kernel. This was a discrete convolution, such that $s^{\prime_{m}}=s_{j}+u_{k}$, $\sigma_{m}^{\prime }=\sigma_{j}\cdot \psi_{k}$, *j *=* *1…*J* (= 16), *k *=* *1…*K* (= 4913), *m *=* *1…*M* (= 16 × 4913 = 78608). For computational speed, the convolved kernel was resampled. A grid of resolution 2 mm was cast over the kernel and the locations, $s^{\prime_{n}}$ of the grid voxels with the highest *N *=* *4*J* (= 64) intensities, $\sigma^{\prime }\prime_{n}$, were selected as the new convolution kernel, with rescaling to ensure conservation of energy. Once the scatter kernel had been convolved with the uncertainty kernel, the resulting kernel was convolved with the primary dose distribution. This was carried out according to eq. (2), but using the resampled scatter kernel:

(3) $$D_{i}\left(r\right)\approx \sum_{n=1}^{N}\Phi \left(r_{i}+{s^{\prime }\prime }_{n}\right){\sigma^{\prime }\prime }_{n},i=1\ldots I,n=1\ldots N.$$

Inverse planning then proceeded using this dose distribution at each iteration. In principle, this could have led to the optimizer attempting to compensate for the uncertainty distribution, but due to the predominantly conformal arcs used, there was not much scope allowed to the optimizer to do this. Consequently, no compensation effects in the form of apertures boosting the periphery of the ITV were observed in the completed plans. The main advantage in using the dose calculation with uncertainty included for the optimization as well as for final dose calculation, was that it avoided a sudden change in dose calculation algorithm at the end of the optimization, which could have limited the quality of the results. For consistency between patients, the same clinical objectives and constraints were used in all cases, as described in Table 1. The importance factors were relative, so that a given dosimetric change in a structure with importance factor 10 had the same effect on the course of the optimization as a dosimetric change 10× larger in a structure with importance factor 1. In the case of probabilistic treatment plans, the PTV objectives were reassigned to the ITV, with the effect that the ITV root‐mean‐square (dose uniformity) objective obtained a total importance of 20. This was to maintain the balance of the optimization between the ITV and the organs at risk.

**Table 1:** Clinical objectives and constraints

Objective	Structure	Statistic	Dose	Importance
Minimize	PTV (ITV)^a^	Root‐mean‐square dose variation with respect to	70 Gy	10
Maximize	PTV (ITV)^a^	Vol irradiated to	54 Gy	10
Minimize	ITV	Root‐mean‐square dose variation with respect to	70 Gy	10
Minimize	Lung‐ITV	Vol irradiated to	11 Gy	4
Minimize	Lung‐ITV	Vol irradiated to	6 Gy	4
Minimize	Heart	Mean dose		1
Minimize	Esophagus	Mean dose		1
Minimize	Proximal airways	Maximum dose		1
Minimize	Spinal cord	Maximum dose		1
Minimize	Spinal cord PRV	Maximum dose		1
Minimize	Brachial plexus	Maximum dose		1
Minimize	Chest wall	Maximum dose		1
Minimize	Skin	Maximum dose		1
Constrain	Spinal cord PRV	Maximum dose	17 Gy	N/A

a when using spatial uncertainty.

## 3 RESULTS

**Figure 2:**
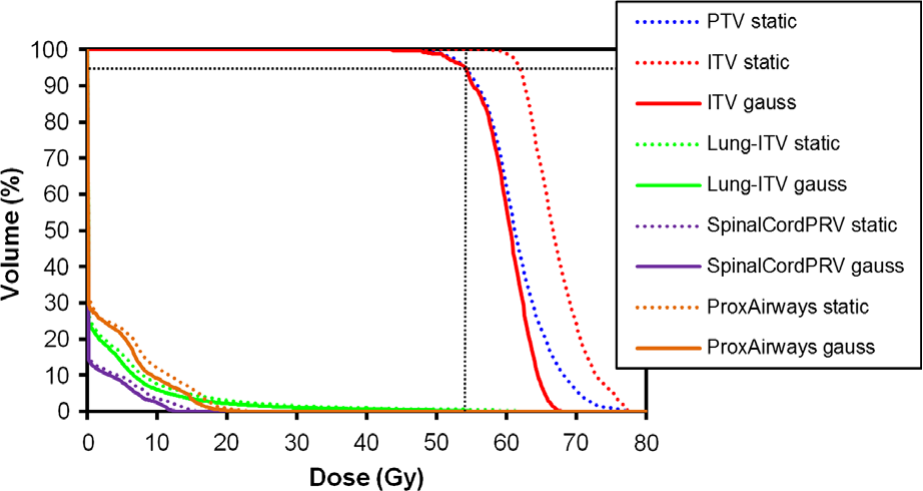
Mean dose–volume histograms for static plans and plans based on a Gaussian distribution of spatial uncertainty. The static plans are prescribed to the PTV *D*_95%_ and the probabilistic plans are prescribed to ITV *D*_95%_.

Mean dose–volume histograms for the static plan and Gaussian uncertainty plan are shown in Fig. 2 to illustrate the overall scenario. For static planning (i.e., without considering uncertainty), the PTV is prescribed such that PTV *D*_95%_ = 54 Gy. Consequently, the ITV receives a much higher dose, with ITV *D*_95%_ ≈ 62 Gy and ITV *D*_50%_ ≈ 67 Gy. If, in contrast, the spatial uncertainty of the ITV is taken into account during inverse planning and dose calculation, the plan can be prescribed such that ITV *D*_95%_ = 54 Gy. In this case the ITV *D*_50%_ ≈ 60 Gy, which is much closer to the intended dose. This is important for the accuracy of radiation delivery to the target, but it also benefits the dose–volume histograms for the organs at risk.

**Figure 3:**
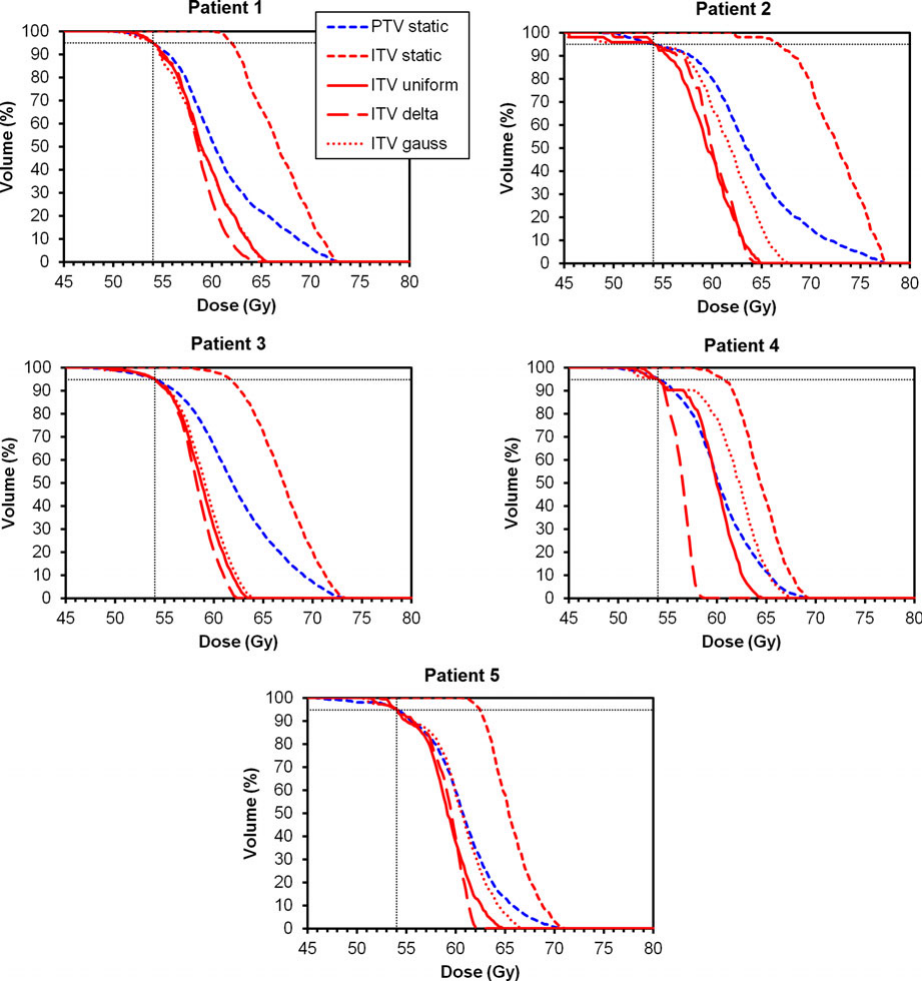
Dose–volume histograms for static and probabilistic plans. The static plans are prescribed to the PTV *D*_95%_ and the probabilistic plans are prescribed to ITV *D*_95%_.

Figure 3 examines this effect in more detail for each patient separately. In all cases, the ITV for static planning receives a much higher dose than the intended prescription dose. When using a probabilistic approach to prescribe to the ITV, the median ITV dose is much closer to the prescribed dose. The choice of uncertainty distribution can be seen from Fig. 3 to have a different impact on each patient, but in general, the delta distribution gives a slightly lower dose than the uniform distribution, and the Gaussian distribution gives a slightly higher dose. Table 2 shows the full dose statistics for all patients. Both the monitor units and ITV *D*_50%_ are lower if the prescription is to the ITV *D*_95%_. This table also indicates that the choice of uncertainty distribution has little effect on the resulting prescription.

**Table 2:** Median ± hemi‐range dose statistics for the five patients in the study

Treatment plan	Static	Uniform	Delta	Gaussian
Fraction monitor units	3109 ± 485	2923 ± 604	2954 ± 463	3016 ± 683
ITV *D*_100%_ (Gy)	60.2 ± 2.8	50.8 ± 2.8	50.8 ± 1.3	49.2 ± 3.4
ITV *D*_99%_ (Gy)	61.0 ± 1.8	51.6 ± 3.5	52.4 ± 1.6	50.8 ± 3.8
ITV *D*_95%_ (Gy)	61.8 ± 2.9	54.0 ± 0.0	54.0 ± 0.0	54.0 ± 0.0
ITV *D*_50%_ (Gy)	66.6 ± 4.2	59.0 ± 0.6	58.4 ± 1.7	60.6 ± 1.7
ITV *D*_50%_ w.r.t. static	–	0.88 ± 0.06	0.88 ± 0.04	0.88 ± 0.06
ITV mean dose (Gy)	66.8 ± 4.0	59.2 ± 0.5	58.5 ± 1.8	60.4 ± 1.4

**Figure 4:**
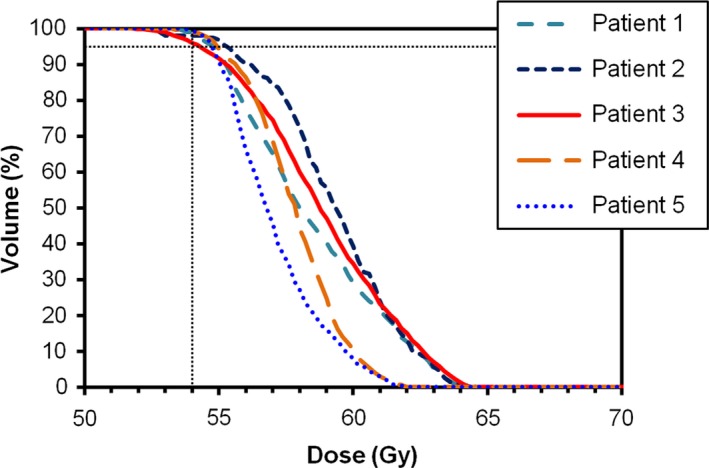
Dose–volume histograms for static plans prescribed to ITV *D*_95%_ and then recalculated using a Gaussian probability distribution.

The result of planning in the conventional manner but prescribing to the ITV *D*_95%_ is shown in Fig. 4. In all cases, when the plan is recalculated including spatial uncertainty with a Gaussian distribution, the ITV *D*_95%_ increases. The magnitude of this increase varies from patient to patient. The statistics for this case are shown in Table 3.

**Table 3:** Median ± hemi‐range dose statistics for a static plan prescribed to ITV *D*_95%_ and evaluated using a Gaussian uncertainty distribution

Treatment plan	Gaussian	Recalculated Gaussian
Fraction monitor units	3016 ± 683	2689 ± 450
ITV *D*_100%_ (Gy)	49.2 ± 3.4	53.2 ± 0.9
ITV *D*_99%_ (Gy)	50.8 ± 3.8	53.8 ± 0.6
ITV *D*_95%_ (Gy)	54.0 ± 0.0	54.8 ± 0.5
ITV *D*_50%_ (Gy)	60.6 ± 1.7	58.0 ± 1.3
ITV *D*_50%_ w.r.t. static	0.88 ± 0.06	0.87 ± 0.04
ITV mean dose (Gy)	60.4 ± 1.4	58.4 ± 1.1

Results using ITV *D*_100%_ (i.e., minimum dose to the ITV) instead of ITV *D*_95%_ are shown in Table 4 for the case of a Gaussian uncertainty distribution. This approach results in a minimum dose to the ITV which is higher than with prescription to the ITV *D*_95%_, but lower than when prescribing to the PTV *D*_95%_. Due to the slightly greater inhomogeneity in ITV dose using this approach (see Fig. 3), the ITV *D*_50%_ is very similar to the static approach. The benefit of this prescription compared to prescribing to ITV *D*_95%_ is that the ITV receives full coverage with 54 Gy, but compared to the static approach, there is little difference. In this case, the uncertainty approach is explicitly modelling a situation similar to that which results from using a PTV and a static approach. The greater dose inhomogeneity in the ITV seen with the uncertainty approach represents the blurring effect of the probability distribution of ITV position.

**Table 4:** Median ± hemi‐range dose statistics for a plan prescribed to ITV *D*_100%_ using a Gaussian uncertainty distribution

Treatment plan	Static	Gaussian
Fraction monitor units	3109 ± 485	4047 ± 745
ITV *D*_100%_ (Gy)	60.2 ± 2.8	54.0 ± 0.0
ITV *D*_99%_ (Gy)	61.0 ± 1.8	55.2 ± 2.0
ITV *D*_95%_ (Gy)	61.8 ± 2.9	59.8 ± 0.9
ITV *D*_50%_ (Gy)	66.6 ± 4.2	67.6 ± 1.2
ITV *D*_50%_ w.r.t. static	–	1.01 ± 0.07
ITV mean dose (Gy)	66.8 ± 4.0	67.4 ± 0.9

## 4 DISCUSSION

The recently published recommendations of ICRU for prescribing and reporting of stereotactic treatments recognize that due to the higher physical density of the ITV compared to the PTV, the ITV may receive a higher dose than that prescribed to the PTV.^5^ Consequently, it is recommended that the ITV *D*_50%_ is reported. This study investigates a further step, which is to prescribe to the ITV while accounting for the uncertainty in its position. This results in an ITV dose which is much closer to the intended prescription. These results are in accord with those of Lacornerie et al.,^29^, ^30^ who also show that the ITV *D*_50%_ is a key statistic for reporting delivered dose.

One possibility for implementation of this technique is to use the spatial uncertainty concept for every patient. However, the results of this study indicate that it may be possible to plan a static treatment, without taking uncertainty into account, based on the PTV but prescribed to the ITV. The resulting ITV *D*_95%_ after recalculation with an uncertainty distribution is not identical to that obtained when planning and prescribing with the uncertainty distribution, but is within 2%. This suggests that a simple re‐prescription is feasible, which would enable a much wider implementation of the findings of this work. In particular, a correction factor describing the impact of the spatial uncertainty on the ITV *D*_95%_ could be used.

In connection with such practical considerations, the study uses a prescription to the ITV *D*_95%_, but other prescriptions are possible. For example, prescription to the ITV *D*_98%_ or *D*_100%_ is also possible, with appropriate adjustment of the prescribed dose, if coverage of the ITV is of concern. Table 4 indicates that there is little difference between prescribing to ITV *D*_100%_ and the static approach in current clinical practice. Similarly, isocentric prescriptions to the ICRU reference point have also been used in the literature.^31^ The results may differ slightly for central tumors, due to the generally higher density of the surrounding tissue in the mediastinal region, but this effect is not expected to alter the conclusion that prescription to the ITV is important.

This work uses a PDF of position to calculate the expected dose distribution in the presence of spatial uncertainty. This is more efficient to calculate and provides a more realistic result than the isocenter shift method, which has also been used in the context of lung radiotherapy.^32^, ^33^ The approach used in this study is to retain the normal calculation of primary fluence but then to use the PDF to model local spatial uncertainty. The uncertainty is considered to be in the ITV position, but the convolution approach implicitly applies the uncertainty distribution throughout the patient, so that other structures are also affected. The magnitude of this effect on other organs is small compared to the change in normal tissue dose shown in Fig. 2 due to change in prescription. The use of a PDF is also closely allied to the field of robust optimization, which has been widely used in proton therapy,^34^, ^35^ where particle range is of particular importance. In this study, the optimizer has only been given limited scope to adapt the treatment plan in compensation for the expected uncertainty. This is due to the predominantly conformal arc.

The work reported in this paper does not address the question of a systematic error in positioning. The uncertainty distributions used are all symmetric about the origin, so assume that a shift is as likely to occur in one direction as the opposite direction. In reality, a contouring error or spatial registration error when registering cone‐beam CT images may result in a shift rather than a symmetrical blurring of the dose distribution.^32^ Use of a worst‐case (minimax) algorithm may enable this to be further investigated.^36^, ^37^, ^38^ This type of algorithm focuses on the impact of the worst‐case scenario with respect to spatial uncertainty, and can therefore better handle a systematic error. Furthermore, although the dose calculation algorithm used in this study is known to be compatible with current commercial convolution algorithms, the results should be validated more thoroughly with a clinical dose calculation algorithm before implementation of the outcome. The dose calculation algorithm described in this study uses a spatially invariant scatter kernel [see eq. (1)], which is known to be less accurate in lung than a full convolution–superposition approach with a variant scatter kernel. This limitation is expected to have a small impact on the results of the study, so validation in a commercial treatment planning system is desirable.

This study shows that the difference between prescription to the PTV *D*_95%_ and ITV *D*_95%_ is of the order of 15%, so that implementation of the results should be carried out with caution, and preferably with the consensus of the international radiation therapy community. A change in this magnitude has already been successfully effected in the context of RTOG 0236 and RTOG 0813, where 60 Gy in 3 fractions without heterogeneity correction is taken as being equivalent to 54 Gy in 3 fractions with heterogeneity corrections.^4^ Although the treatments are ablative in intent, so that it may be argued that the minimum dose is the most important, it is nonetheless important that delivered doses are accurately prescribed and reported. This study proposes a method of taking a further step toward this goal.

## 5 CONCLUSION

This study shows that using a robust probabilistic approach to planning SBRT lung treatments, and prescribing to the ITV, results in the ITV receiving a dose closer to the intended prescription than when using a static approach and prescription to the PTV. The exact form of the uncertainty distribution used in this approach is not found to be critical. Planning the treatment with a static approach and prescribing to the ITV gives a result which is very close to that obtained with a probabilistic approach.

## CONFLICT OF INTEREST

The authors declare no conflict of interest.
